# Author Correction: *Pogz* deficiency leads to transcription dysregulation and impaired cerebellar activity underlying autism-like behavior in mice

**DOI:** 10.1038/s41467-021-24166-w

**Published:** 2021-06-17

**Authors:** Reut Suliman-Lavie, Ben Title, Yahel Cohen, Nanako Hamada, Maayan Tal, Nitzan Tal, Galya Monderer-Rothkoff, Bjorg Gudmundsdottir, Kristbjorn O. Gudmundsson, Jonathan R. Keller, Guo-Jen Huang, Koh-ichi Nagata, Yosef Yarom, Sagiv Shifman

**Affiliations:** 1 Department of Genetics, The Institute of Life Sciences, The Hebrew University of Jerusalem, Jerusalem, Israel; 2 Department of Neurobiology, The Institute of Life Sciences and Edmond & Lily Safra Center for Brain Sciences (ELSC), The Hebrew University of Jerusalem, Jerusalem, Israel; 3 Department of Molecular Neurobiology, Institute for Developmental Research, Aichi Developmental Disability Center, Kasugai, Japan; 4 Cellular and Molecular Therapeutics Branch, National Heart Lung and Blood Institutes (NHLBI)/National Institute of Diabetes and Digestive and Kidney Diseases (NIDDK), National Institutes of Health (NIH), Bethesda, MD USA; 5 Mouse Cancer Genetics Program, Center for Cancer Research, National Cancer Institute at Frederick, Bldg. 560/12-70, 1050 Boyles Street, Frederick, MD 21702 USA; 6 Basic Research Program, Leidos Biomedical Research Inc, Frederick National Laboratory for Cancer Research, Bldg. 560/32-31D, 1050 Boyles Street, Frederick, MD 21702 USA; 7 Department and Graduate Institute of Biomedical Sciences, College of Medicine, Chang Gung University, Taoyuan, Taiwan; 8 Department of Neurochemistry, Nagoya University Graduate School of Medicine, Nagoya, Japan

**Keywords:** Gene expression, Genomics, Autism spectrum disorders, Gene expression, Genomics, Autism spectrum disorders

Correction to: *Nature Communications* 10.1038/s41467-020-19577-0, published online 17 November 2020.

The original version of the Supplementary Information associated with this Article omitted Table S3 and Table S4 due to a technical error. The HTML has been updated to include Table S3 and Table S4 as Supplementary Data associated with this Correction.

## Supplementary information

Supplementary Table S3

Supplementary Table S4

