# Supplementary material for: Pogz deficiency leads to transcription dysregulation and impaired cerebellar activity underlying autism-like behavior in mice
Source: Nat Commun. 2020 Nov 17;11:5836. doi: 10.1038/s41467-020-19577-0 (PMC7673123; doi:10.1038/s41467-020-19577-0)
Supplement: Supplementary file 1 — Supplementary Information [file 41467_2020_19577_MOESM1_ESM.pdf]

Pogz deficiency leads to transcription dysregulation and impaired cerebellar activity underlying autism-like behavior in mice

Reut Suliman, Ben Title, Yahel Cohen, Nanako Hamada, Maayan Tal, Nitzan Tal, Galya Monderer-Rothkoff, Bjorg Gudmundsdottir, Kristbjorn O. Gudmundsson, Jonathan R Keller, Guo-Jen Huang, Koh-ichi Nagata, Yosef Yarom and Sagiv Shifman

Supplementary Information

Figures S1-7, Table S1-S2

Figure S1

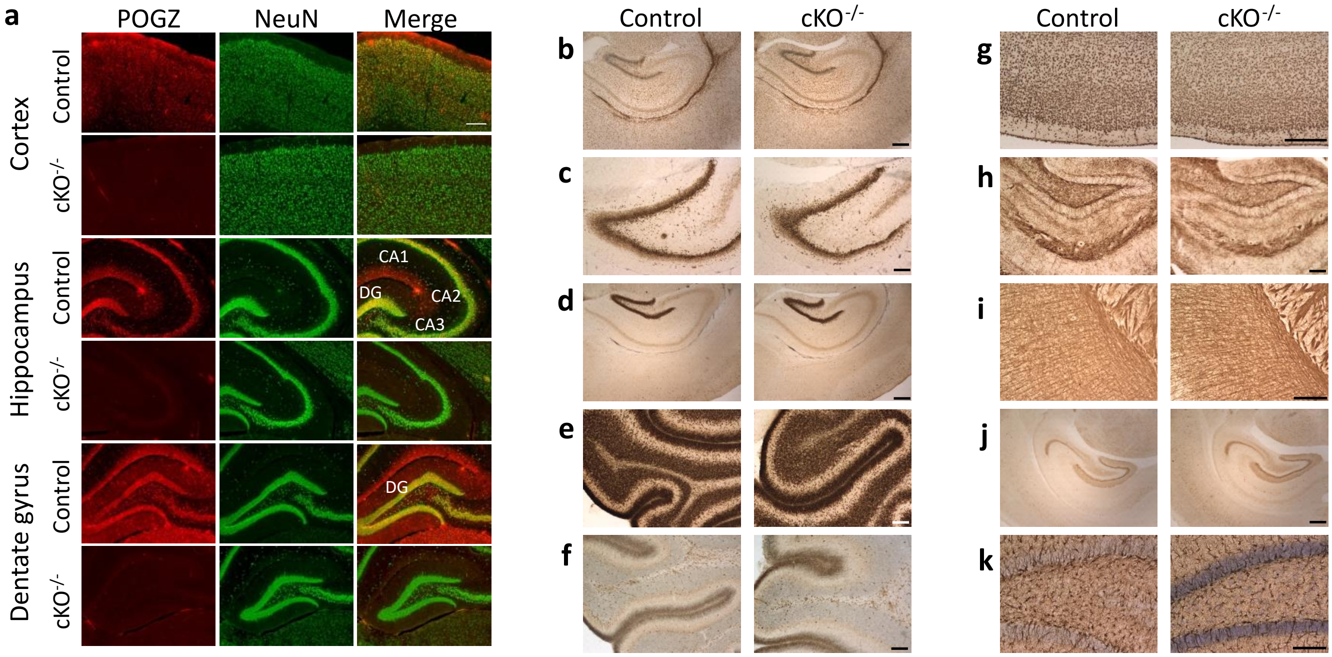

Supplementary Figure 1. (A) Immunofluorescence staining of control and *Pogz* cKO$^{-/-}$ mice brains (P60) using antibodies against POGZ (red) and NeuN (green) showing a widespread expression of POGZ in the control mice brains (cortex, hippocampus, dentate gyrus) and its absence from *Pogz* cKO$^{-/-}$ mice brains. n = 3 control, 3 cKO$^{-/-}$. Scale bar = 100µm. (B-K) Immunohistochemistry staining for various neuronal markers showing that *Pogz* cKO$^{-/-}$ mice do not display any detectable anatomical defects (B) SOX2 marker for adult neural stem cells. Scale bar = 250µm (C) NeuroD marker for differentiating of hippocampal neurons. Scale bar = 100µm (D) PROX1 marker for the DG. Scale bar = 250µm (E) PAX6 marker for the cerebellar granular layer. Scale bar = 100µm (F) Ki67 marker for proliferation. Scale bar = 100µm. (B-F) were done on mouse developing brains (P11). (G) CUX1 marker for specific cortical layers (II-IV). Scale bar = 250µm. (H-I) CNPase marker for oligodendrocytes and Schwann cells. Scale bar = 100µm (J) Calbindin marker for GABAergic interneurons. Scale bar = 250µm (K) GFAP marker for developing astrocytes and ependymal cells. For B-K, n = 3 control, 3 cKO$^{-/-}$ in one independent experiment. Scale bar = 100µm.

Figure S2

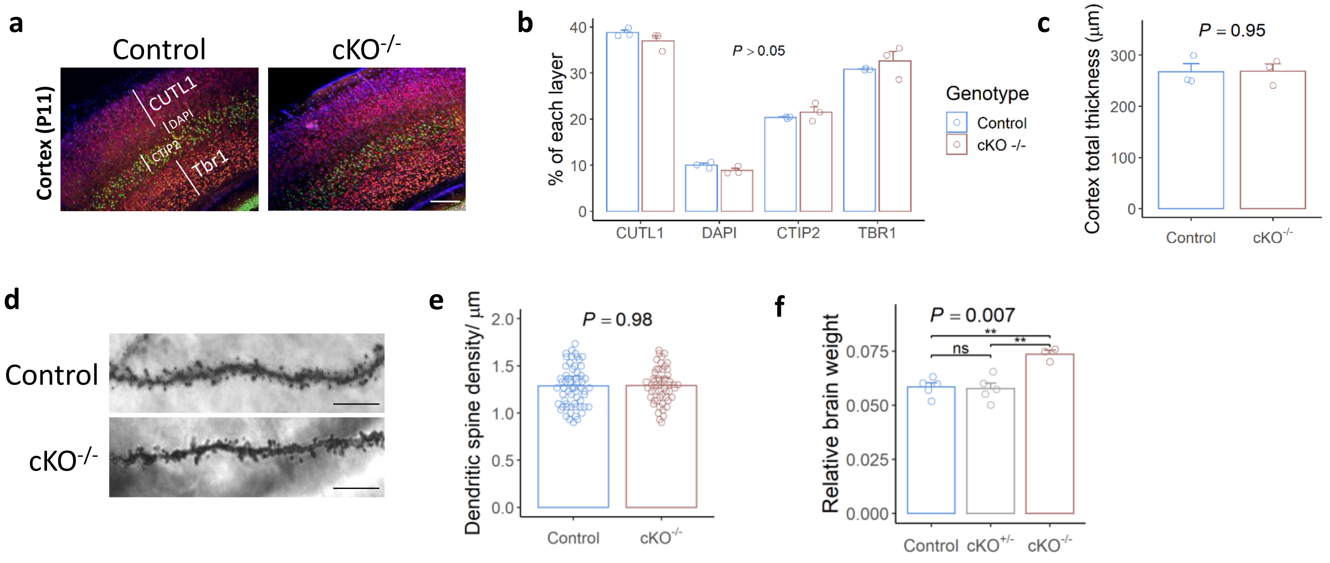

Supplementary Figure 2. (A) Immunostaining for specific cortical layers with known markers (CUTL1, CTIP2, Tbr1) and DAPI at P11. Scale bar = 100µm. (B) Quantification of the relative thickness of each cortical layer was based on the length of layers stained with the markers or stained only by DAPI. n= 3 control (blue), 3 cKO$^{-/-}$ (red); all *P* > 0.05, Two-tailed t-test. (C) Quantification of the total thickness of the cortex. Two-tailed t-test. (D-E) Dendritic spine density in the dentate gyrus (DG) of control and cKO$^{-/-}$ mice (D) Representative image used for dendritic spines density analysis in the dentate gyrus (DG). Spines were measured at least 40µm far from the soma, on a 30µm dendrite length and were counted manually using imageJ software. Scale bar = 5µm (E) Quantification of dendritic spines density. n {animals/dendrites} = 3/59 control, 3/52 cKO$^{-/-}$, Two-tailed t-test. (F) Relative brain weight (brain weight/body weight) at P11. n = 5 control (blue), 5 cKO$^{+/-}$ (grey), and 3 cKO$^{-/-}$ (red). The *P*-values at the top of the plot are for association between the quantitative measurements and the number of intact *Pogz* alleles calculated with a linear regression model. Pairwise comparisons between genotypes was calculated by two-tailed t-test and the significance is represented by: **, P < 0.01; ns, not significant. Quantitative data are mean ± SEM**.**

Figure S3

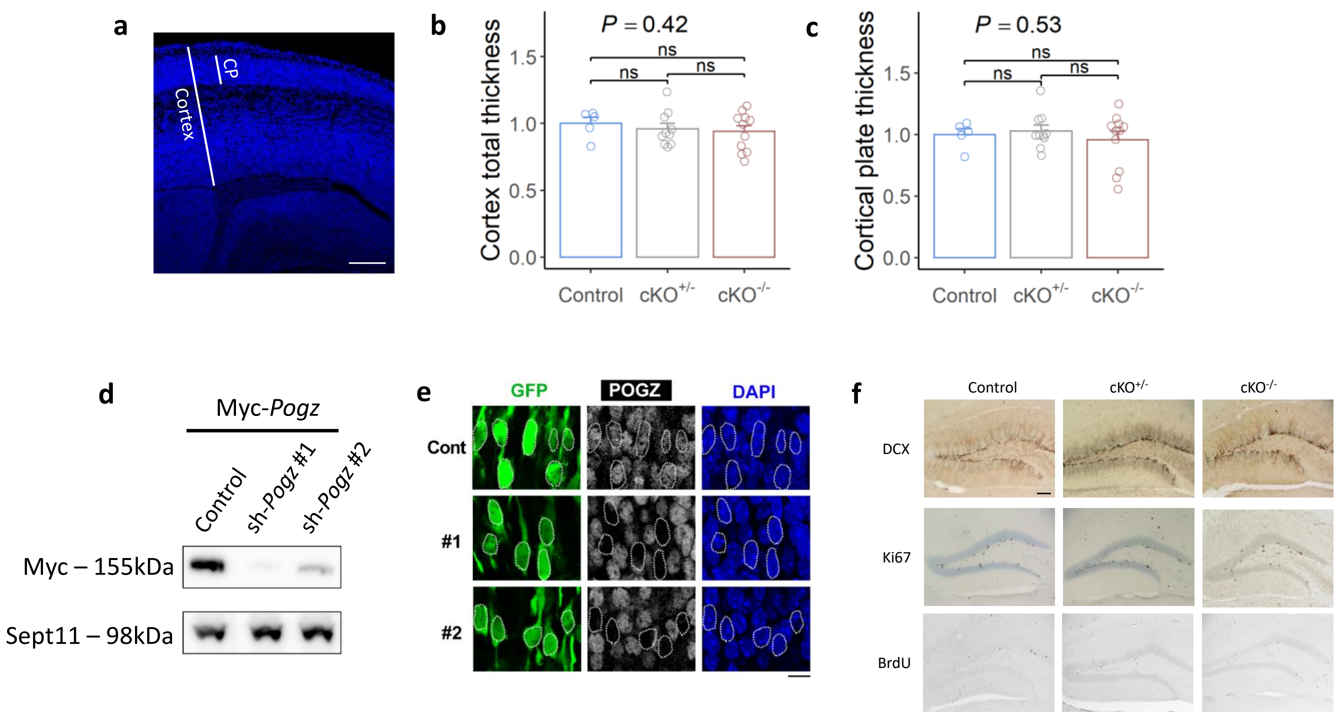

Supplementary Figure 3. (A) A representative image of the areas measured for the cortex total thickness and the thickness of the cortical plate (CP). Scale bar = 200µm (B-C) Quantification of the total thickness of the cortex (B) and the cortical plate thickness (C) (E15.5). Data is relative to control mice. n = 5 control (blue), 10 cKO$^{+/-}$ (grey), 11 cKO$^{-/-}$ (blue) in one independent experiment. The *P*-values at the top of the plots are for association between the quantitative measurements and the number of intact *Pogz* alleles calculated with a linear regression model. Pairwise comparisons between genotypes was calculated by t-test and the significance is represented by: ns, not significant. (D) Characterization of *Pogz*-RNAi vectors. pCAG-Myc-Pogz (0.2 µg) was transfected into COS7 cells with pSuper-H1.shLuc (Control), sh-Pogz#1 or #2 (1.0 µg each). After 48 h, cells were harvested and subjected to western blotting with anti-Myc (upper panel). Anti-Sept11 was used for a loading control (lower panel). One independent experiment (E) Knockdown of endogenous POGZ in migrating cortical neurons. pCAG-EGFP (0.5 µg) was electroporated in utero with sh-Pogz#1 or #2 (1.0 µg each) into E14.5 embryonic brains. Cortical slices were prepared and fixed at P0. Cells were then immunostained with anti-GFP (green), anti-POGZ (white) and DAPI (blue). GFP-positive cells were marked with dotted lines. Scale bar = 10 µm. (F) Representative Immunostaining images (positive DAB color) of the adult dentate gyrus used for quantification of DCX, Ki67 and BrdU. n = 6 control, 6 cKO$^{+/-}$, 3 cKO$^{-/-}$ in two independent experiments. Scale bar = 100µm. Quantitative data are mean ± SEM.

Supplementary Table 1. Summary of behavioral assays and their results

Phenotypes	Test	Result
Growth delay	Body weight	Decreased
Microcephaly	Brain weight	Decreased
Motor coordination	Rotarod	Deficits
	Horizontal bar	Deficits
General activity	Open field	NS
Working memory	T-maze	Deficits
Spatial learning & memory	Morris water maze	Deficits
Anxiety	Elevated plus maze	NS
	Open field	NS
Repetitive behavior	Grooming	NS
	Marble burying	Decreased
Social behavior	Social approach	Increased
	Social novelty	NS
	Direct social interactions	Increased
	Olfactory habituation/dishabituation task	Increased sniffing

Figure S4

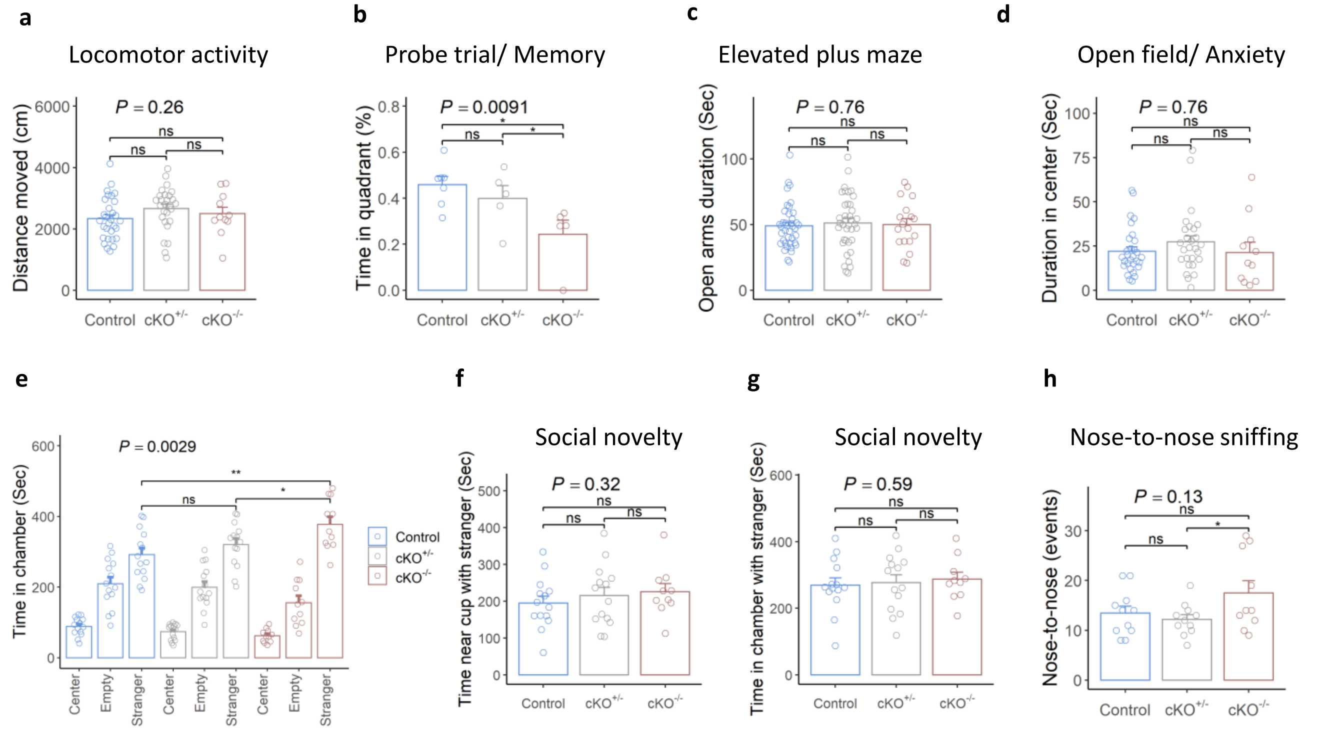

Supplementary Figure 4. *Pogz*-deficient mice show abnormal motor cognitive, and social behavior (A) Total distance moved in the open field. n= 30 control (blue), 28 $cKO^{+/-}$ (grey), 11 $cKO^{-/-}$ (red). (B) Memory performance was assessed in a probe test based on the proportion of time in the north east quadrant that contained the escape platform in the training trials controlled for age. n= 7 control, 5 $cKO^{+/-}$, 5 $cKO^{-/-}$. (C) Time spent in the open arms of the elevated plus maze. n= 40 control, 38 $cKO^{+/-}$, 18 $cKO^{-/-}$. (D) Duration in center of the open field, n= 30 control, 27 $cKO^{+/-}$, 11 $cKO^{-/-}$. (E) Sociability assessed by the three chambers test. Time spent in each of the three chambers (center, empty chamber, and chamber with a stranger mouse). n= 14 control, 14 $cKO^{+/-}$, 11 $cKO^{-/-}$. (F) Time spent in the chamber and (G) near the cup of the stranger mouse during the social novelty test. n= 14 control, 14 $cKO^{+/-}$, 11 $cKO^{-/-}$. (H) The number of nose-to-nose sniffing as measured in the direct social interaction test. n= 11 control, 11 $cKO^{+/-}$, 10 $cKO^{-/-}$. The *P*-values at the top of the plots are for association between the quantitative measurements and the number of intact *Pogz* alleles calculated with a linear regression model (unless stated otherwise). Pairwise comparisons between genotypes was calculated by two-tailed t-test and the significance is represented by: *, $P < 0.05$; **, $P < 0.01$; ns, not significant. Quantitative data are mean ± SEM.

Supplementary Table 2. The table shows the Akaike information criterion (AIC) values for three genetic model. A lower value means a better fit of the model. The minimal values are labeled by color.

Akaike information criterion (AIC)			
Trait	Additive	Dominant	Recessive
Horizontal bar	133.2	139.9	130.3
Marble burying	180.1	187.3	180.2
T-maze	157.4	159.7	157.5
Social approach	442.6	445.2	444.0
Social interactions	281.7	282.3	284.0
Anogenital sniffing	200.5	205.7	197.4

Figure S5

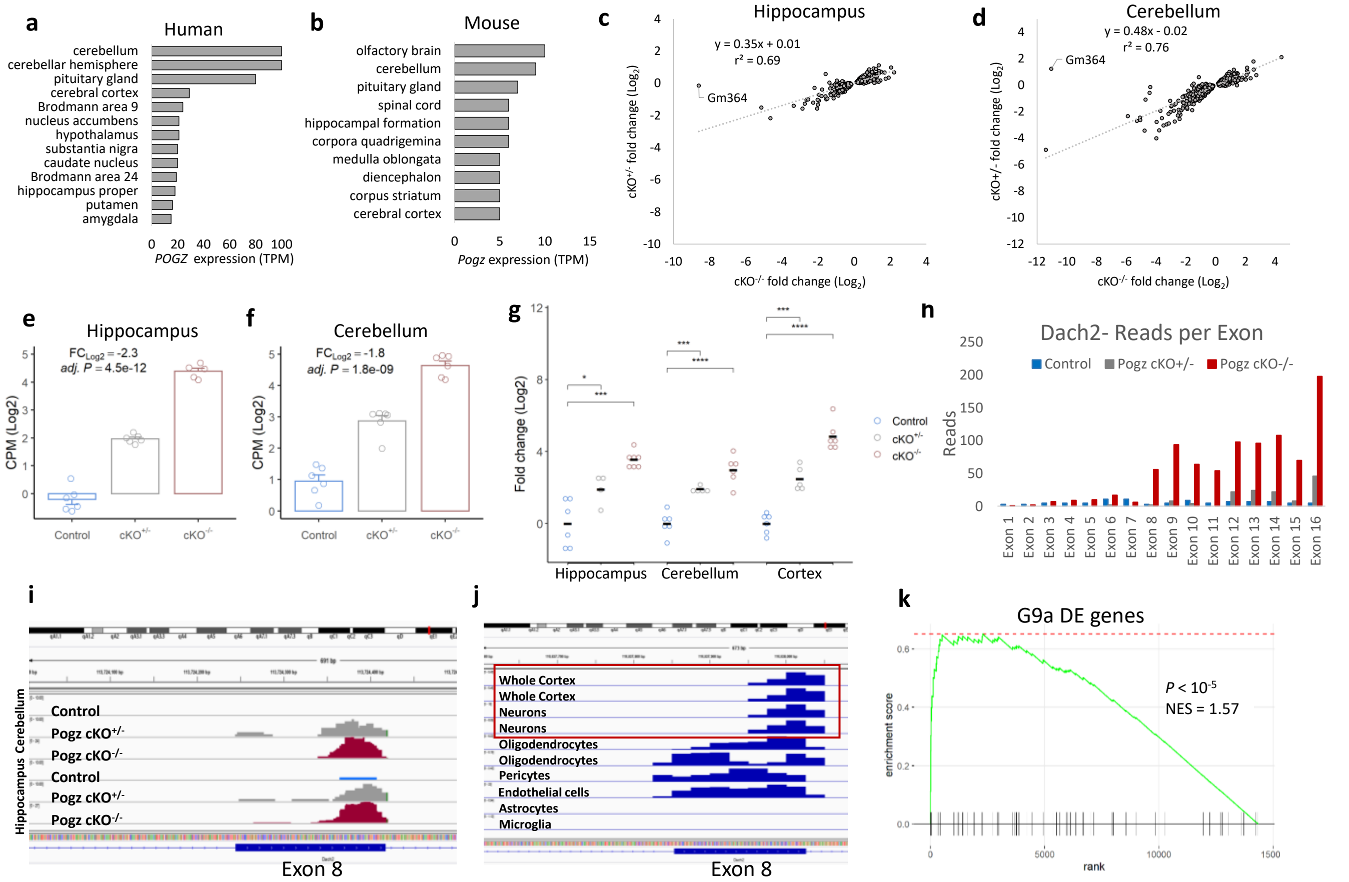

Supplementary Figure 5. *Pogz* deficiency leads to transcriptional dysregulation (A) Levels of *POGZ* expression in different brain areas of human and (B) in mouse (FANTOM5 dataset). Values are transcripts per million (TPM) (C-D) Additive effect of *Pogz* on gene expression in (C) hippocampus and (D) cerebellum. The fold change (Log2) in gene expression of $cKO^{+/-}$ is plotted against the fold change of $cKO^{-/-}$. The fold change is based on pairwise analysis of the RNA-seq data, and is shown for genes with FDR<5%. Gm364 is an outlier that shows a recessive effect (upregulated only in $cKO^{-/-}$). (E-F) *Dach2* expression levels from RNA-Seq in the (E) hippocampus and (F) cerebellum. n= 6 control, 6 cKO+/-, 6 $cKO^{-/-}$. Quantitative data are mean ± SEM. CPM, counts per million; FCLog2, Log2 of the average fold change; adj.P, *P*-value calculated by edgeR and adjusted for multiple testing by FDR procedure. (G) The relative expression of *Dach2* in qPCR experiment with three brain regions (Hippocampus, cerebellum and cortex). The black horizontal line indicates the mean. Two-tailed t-test; *, P < 0.05; ***, P < 0.001; ****, P < 0.0001. (H-J) *Dach2* has a shorter and unique isoform in neurons (H) Reads per exon for *Dach2* in the cerebellum showing an increase in the number of reads from Exon 8 to 16 in *Pogz*-deficient mice (I) Enlargement of Exon 8, showing reads that are mapped mainly to the end of the exon, both in the hippocampus and cerebellum. (J) Similar distribution of *Dach2* reads in neurons and whole cortex relative to other brain cells (Oligodendrocytes, Endothelial cells, Microglia and Pericytes). Data from: http://jiaqianwulab.org/braincell/RNASeq.html. (K) Genes differentially expressed in the G9a mouse model are significantly enriched among genes differentially expressed in the Pogz mouse model. The significance of the enrichment was calculated using fgseaMultilevel function in the 'fgsea Package' in R.

Figure S6

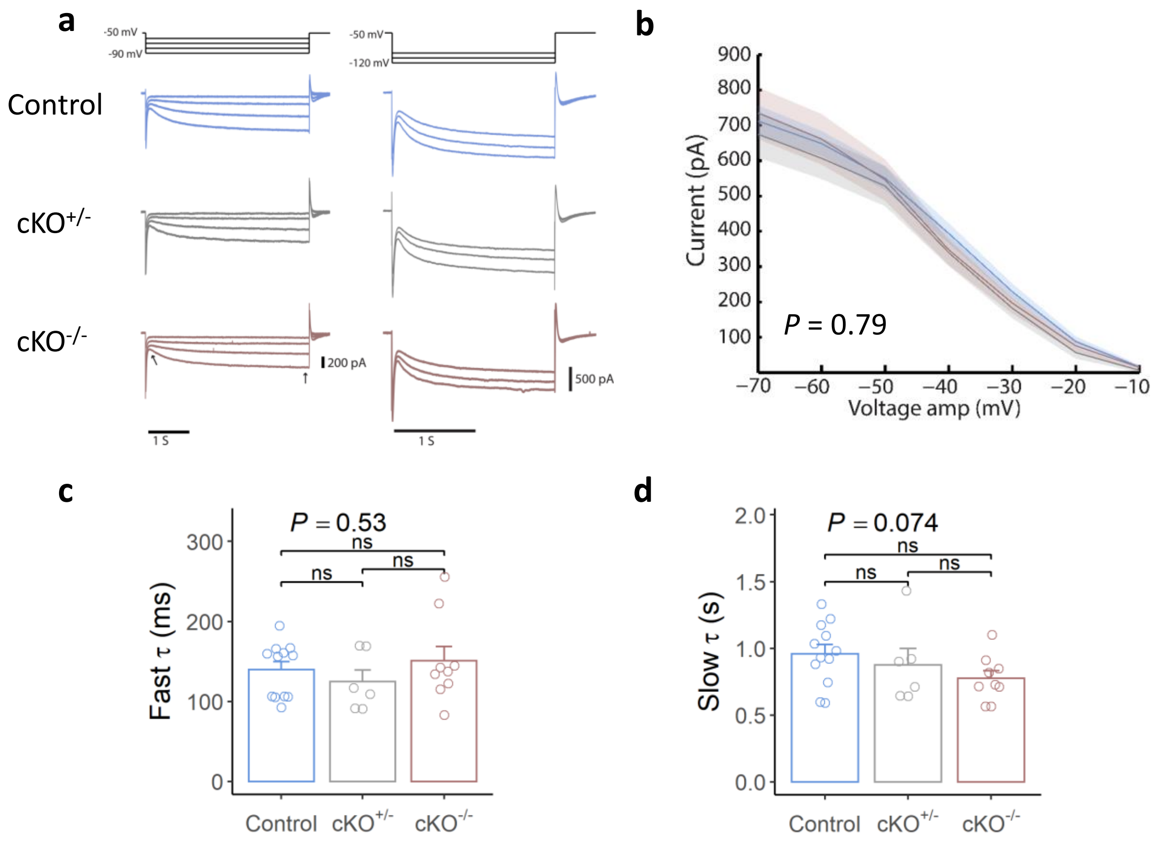

Supplementary Figure 6. I_h current in Purkinje cells is unaffected by *Pogz***.** (A) Representative examples of current traces evoked by negative voltage steps from a holding potential of -50mV (*left*: 4 sec, *right*: 2 sec). Left arrow (bottom left trace) indicates the initial current response, right arrow indicates the steady-state current. (B) Comparison of the voltage dependence of the I_h current in all genotypes. Repeated-measures ANOVA. Fast (C) and slow (D) I_h kinetics of all genotypes, as measured by fitting a double exponent to traces (see Material and methods). n = 12/3 control (blue), 6/1 cKO$^{+/-}$ (grey), 9/2 cKO$^{-/-}$ (grey). The *P*-values at the top of the plots are for association between the quantitative measurements and the number of intact *Pogz* alleles calculated with a linear regression model (unless stated otherwise). Pairwise comparisons between genotypes was calculated by two-tailed t-test. ns, not significant. Quantitative data are mean ± SEM.

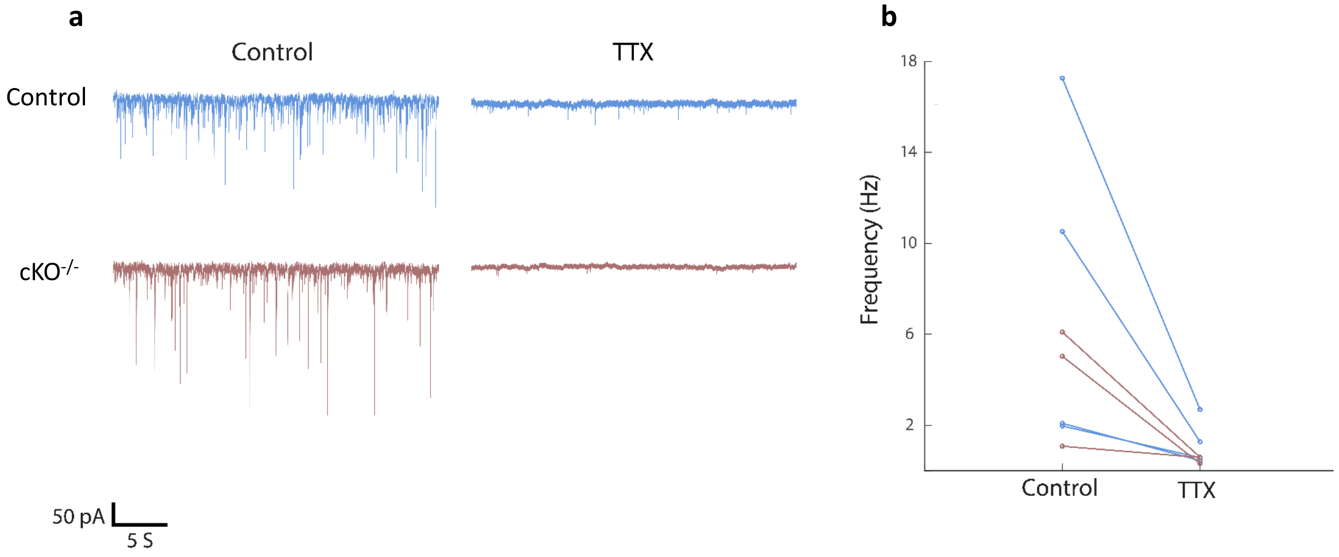

Supplementary Figure 7. The spontaneous IPSCs in PCs, in control and *Pogz* deficient mice, are mostly generated by presynaptic action potentials. (A) Continuous recordings of sIPSCs and mIPSCs recorded from PCs of control and $cKO^{-/-}$. (B) Application of Tetrodotoxin (TTX, 1µM) remarkably reduced IPSC frequency in all of the recorded cells of both control and $cKO^{-/-}$ mice.
