# Supplementary material for: Pogz deficiency leads to transcription dysregulation and impaired cerebellar activity underlying autism-like behavior in mice
Source: Nat Commun. 2020 Nov 17;11:5836. doi: 10.1038/s41467-020-19577-0 (PMC7673123; doi:10.1038/s41467-020-19577-0)
Supplement: Supplementary file 3 — Reporting Summary [file 41467_2020_19577_MOESM3_ESM.pdf]

nature research

Corresponding author(s): Yossef Yarom and Sagiv Shifman

Last updated by author(s): Oct 1, 2020

Reporting Summary

Nature Research wishes to improve the reproducibility of the work that we publish. This form provides structure for consistency and transparency in reporting. For further information on Nature Research policies, see our Editorial Policies and the Editorial Policy Checklist.

Statistics

For all statistical analyses, confirm that the following items are present in the figure legend, table legend, main text, or Methods section.

n/a	Confirmed	
☐	☒	The exact sample size (*n*) for each experimental group/condition, given as a discrete number and unit of measurement
☐	☒	A statement on whether measurements were taken from distinct samples or whether the same sample was measured repeatedly
☐	☒	The statistical test(s) used AND whether they are one- or two-sided<br>*Only common tests should be described solely by name; describe more complex techniques in the Methods section.*
☐	☒	A description of all covariates tested
☐	☒	A description of any assumptions or corrections, such as tests of normality and adjustment for multiple comparisons
☐	☒	A full description of the statistical parameters including central tendency (e.g. means) or other basic estimates (e.g. regression coefficient) AND variation (e.g. standard deviation) or associated estimates of uncertainty (e.g. confidence intervals)
☐	☒	For null hypothesis testing, the test statistic (e.g. *F*, *t*, *r*) with confidence intervals, effect sizes, degrees of freedom and *P* value noted<br>*Give P values as exact values whenever suitable.*
☒	☐	For Bayesian analysis, information on the choice of priors and Markov chain Monte Carlo settings
☒	☐	For hierarchical and complex designs, identification of the appropriate level for tests and full reporting of outcomes
☒	☐	Estimates of effect sizes (e.g. Cohen's *d*, Pearson's *r*), indicating how they were calculated

Our web collection on statistics for biologists contains articles on many of the points above.

Software and code

Policy information about availability of computer code

Data collection	Whole Cell Recordings: A dedicated software based on the LabView platform (National Instruments, version 15.0) was used for monitoring and data acquisition. Animal behavior: Noldus Ethovision XT11 software. RNA seq: STAR aligner (version 2.5).
Data analysis	Statistical analysis was performed using common functions in R (3.6.1). Analysis of whole cell and in-vivo extracellular PC recordings was using Matlab (2018a) (Mathworks, Natick, Ma, USA).

For manuscripts utilizing custom algorithms or software that are central to the research but not yet described in published literature, software must be made available to editors and reviewers. We strongly encourage code deposition in a community repository (e.g. GitHub). See the Nature Research guidelines for submitting code & software for further information.

Data

Policy information about availability of data

All manuscripts must include a data availability statement. This statement should provide the following information, where applicable:

- Accession codes, unique identifiers, or web links for publicly available datasets
- A list of figures that have associated raw data
- A description of any restrictions on data availability

The RNA-Seq data have been deposited to a public database (GEO accession 144648). All other data supporting the findings of this study are available within the paper and its supplementary information files. Raw data is available for all figures.

Field-specific reporting

Please select the one below that is the best fit for your research. If you are not sure, read the appropriate sections before making your selection.

- [x] Life sciences
- [ ] Behavioural & social sciences
- [ ] Ecological, evolutionary & environmental sciences

For a reference copy of the document with all sections, see nature.com/documents/nr-reporting-summary-flat.pdf

Life sciences study design

All studies must disclose on these points even when the disclosure is negative.

Sample size	We did not use statistical methods to predetermine the sample sizes, but the sample sizes in this study are similar to those generally employed in this field.
Data exclusions	In the RNA-seq an outlier RNA sample was excluded based on Multi-Dimensional Scaling Plot and other diagnosis plots that indicated that the sequencing was abnormal. This exclusion criteria was not pre-established. For the behavioral assays non-responding animals were excluded.
Replication	All experiments had biological replicates with similar results. We included detailed numbers in the manuscript.
Randomization	Genotypes were randomly allocated for the different assays.
Blinding	All histological, cell biological, behavioral and electrophysiological experiments were performed blinded to genotypes.

Reporting for specific materials, systems and methods

We require information from authors about some types of materials, experimental systems and methods used in many studies. Here, indicate whether each material, system or method listed is relevant to your study. If you are not sure if a list item applies to your research, read the appropriate section before selecting a response.

Materials & experimental systems

n/a	Involved in the study	
☐	☒	Antibodies
☐	☒	Eukaryotic cell lines
☒	☐	Palaeontology and archaeology
☐	☒	Animals and other organisms
☒	☐	Human research participants
☒	☐	Clinical data
☒	☐	Dual use research of concern

Methods

n/a	Involved in the study	
☒	☐	ChIP-seq
☒	☐	Flow cytometry
☒	☐	MRI-based neuroimaging

Antibodies

Antibodies used	IHC and IF: Rabbit anti POGZ (ab171934 1:500); Mouse anti NeuN (MAB377 1:400); Mouse anti Anti-CUTL1/CUX1 (ab54583 1:500); Rat anti CTIP2 (ab18465 1:500); Rabbit anti TBR1 (Merck AB10554 1:400); Rabbit anti Ki67 (ab15580 1:1000); Rat anti BrdU (ab6326 1:250) Mouse anti DCX (sc271390 1:200); Mouse anti SOX2 (sc17320 1:1000); Goat anti NeuroD (sc1804 1:1000); Rabbit anti PROX1 (Merck AB5475 1:1000); Rabbit anti PAX6 (Merck AB2237 1:500); Mouse anti CNPase (ab6319 1:1000); Mouse anti Calbindin (Merck C9848 1:1000); Rabbit anti GFAP (ab7260 1:1000); goat anti rabbit Alexa fluor 568 (Invitrogen A-11011 1:200); goat anti mouse Alexa fluor 488 (Invitrogen A-11001 1:200). Goat anti Rabbit IgG biotinylated (Vector labs BA-1000 1:250); Rabbit anti Goat IgG biotinylated (Vector labs BA-5000 1:250); Rabbit anti Rat IgG biotinylated (Vector labs BA-4000 1:250); Horse anti Mouse IgG biotinylated (Vector labs BA-2000 1:250)<br>Staining of embryonic brain slices: Anti POGZ was prepared as described26; Chicken anti GFP (Aves Laboratories GFP-1020 1:1000); Rabbit anti-Ki67 (Thermo Scientific RB-1510 1:1000); Rabbitanti-Pax6 (BioLegend PRB 278P 1:500); Rabbit anti TBR2 (ab23345 1:250); Rabbit anti phospho-histon H3 (Cell Signaling Technology 9701 1:400) Alexa Fluor® 488-labeled IgG was used as secondary antibody (ab150077 1:200).<br>WB Primary antibodies: Rabbit anti POGZ (ab167408 1:500); Rat anti βTubulin (ab6160 1:10000); Rabbit polyclonal antibodies against Myc-tag (1:300) and a cytoskeletal protein, Sept11 (1:1000), were produced as described73,74. WB Secondary abs: Donkey anti Rabbit IgG HRP (ab7083 1:10000); Donkey anti Rat IgG HRP (ab102265 1:10000).
Validation	The antibodies were purchased from qualified vendors that provide validation on the website of the manufacturer

Eukaryotic cell lines

Policy information about cell lines

Cell line source(s)	N2a (ATCC® CCL-131™), 293T (ATCC® CRL-3216™) and Cos7 (ATCC® CRL-1651™) cells were obtained from ATCC
Authentication	None
Mycoplasma contamination	All cell lines used are confirmed mycoplasma free (by ATCC)
Commonly misidentified lines (See ICLAC register)	No commonly misidentified cell lines were used

Animals and other organisms

Policy information about studies involving animals; ARRIVE guidelines recommended for reporting animal research

Laboratory animals	C57/BL6 males and females at 8-16 weeks of age. Mice were group-housed in a room with a 12-h-light, 12-h-dark cycle (lights on at 7:00) in 20-22°C (humidity 55±10%) with access to food and water ad libitum.
Wild animals	The study did not involve wild animals
Field-collected samples	The study did not involve samples collected from the field
Ethics oversight	All mouse studies were approved by the Institutional Animal Care and Use Committees at The Hebrew University of Jerusalem.

Note that full information on the approval of the study protocol must also be provided in the manuscript.
